# Comparison of same day diagnostic tools including Gene Xpert and unstimulated IFN-γ for the evaluation of pleural tuberculosis: a prospective cohort study

**DOI:** 10.1186/1471-2466-14-58

**Published:** 2014-04-08

**Authors:** Richard Meldau, Jonny Peter, Grant Theron, Greg Calligaro, Brian Allwood, Greg Symons, Hoosain Khalfey, Gina Ntombenhle, Ureshnie Govender, Anke Binder, Richard van Zyl-Smit, Keertan Dheda

**Affiliations:** 1 Lung Infection and Immunity Unit, Division of Pulmonology & UCT Lung Institute, Department of Medicine, University of Cape Town, Cape Town, South Africa; 2 Institute of Infectious Diseases and Molecular Medicine, University of Cape Town, Cape Town, South Africa

**Keywords:** Tuberculosis, Diagnosis, Xpert MTB/RIF, Interferon gamma, Adenosine deaminase, Pleural fluid

## Abstract

**Background:**

The accuracy of currently available same-day diagnostic tools (smear microscopy and conventional nucleic acid amplification tests) for pleural tuberculosis (TB) is sub-optimal. Newer technologies may offer improved detection.

**Methods:**

Smear-microscopy, adenosine deaminase (ADA), interferon gamma (IFN-γ), and Xpert MTB/RIF [using an unprocessed (1 ml) and centrifuged (~20 ml) sample] test accuracy was evaluated in pleural fluid from 103 consecutive patients with suspected pleural TB. Culture for *M.tuberculosis* and/or histopathology (pleural biopsy) served as the reference standard. Patients were followed prospectively to determine their diagnostic categorisation.

**Results:**

Of 93 evaluable participants, 40 had definite-TB (reference positive), 5 probable-TB (not definite but treated for TB) and 48 non-TB (culture and histology negative, and not treated for TB). Xpert MTB/RIF sensitivity and specificity (95% CI) was 22.5% (12.4 - 37.6) and 98% (89.2 - 99.7), respectively, and centrifugation did not improve sensitivity (23.7%). The Xpert MTB/RIF internal positive control showed no evidence of inhibition. Biomarker specific sensitivity, specificity, PPV, and NPVs were: ADA (48.85 IU/L; rule-in cut-point) 55.3% (39.8 - 69.9), 95.2% (83.9 - 98.7), 91.4 (73.4 - 95.4), 69.7% (56.7 - 80.1); ADA (30 IU/L; clinically used cut-point) 79% (63.7 - 89), 92.7% (80.6 - 97.5), 91.0 (73.4 - 95.4), 82.7% (69.3 - 90.1); and IFN-γ (107.7 pg/ml; rule-in cut-point) 92.5% (80.2 - 97.5), 95.9% (86.1 - 98.9), 94.9% (83.2 - 98.6), 93.9% (83.5 - 97.9), respectively (IFN-γ sensitivity and NPV better than Xpert [p < 0.05] and rule-in ADA [p < 0.05]).

**Conclusion:**

The usefulness of Xpert MTB/RIF to diagnose pleural TB is limited by its poor sensitivity. IFN-γ is an excellent rule-in test and, compared to ADA, has significantly better sensitivity and rule-out value in a TB-endemic setting.

## Background

Tuberculosis (TB) remains a global health problem, with an estimated 1.4 million deaths and 8.7 million new cases reported in 2011 [1]. Pulmonary TB is the most common form of TB, with extrapulmonary TB (EPTB) accounting for ~15% of cases, but this estimate increases to ~50% in high HIV prevalence settings [2]. Pleural TB, a common form of EPTB, remains a common problem for physicians practising in high, intermediate and low TB burden settings, particularly where there are large immigrant populations. The diagnosis of pleural TB is challenging due to the paucibacillary nature of biological samples, and the need for diagnostic confirmation using invasive, expensive, and time consuming procedures such as blind pleural biopsy, imaging-guided-pleural biopsy, and medical or surgical thoracoscopy [3].

Proxy markers such as adenosine deaminase (ADA), an enzyme that catalyzes the conversion of adenosine and deoxyadenosine to inosine and deoxyinosine has been widely studied [4]. ADA testing is relatively easy, inexpensive and rapid, with pooled sensitivity and specificity estimates of 92% and 90%, respectively, across different prevalence settings depending on the cut-point used [4]. Interferon gamma (IFN-γ), an inflammatory cytokine secreted from macrophages and CD4 (+) T cells in response to *M. tuberculosis* infection, and that tends to concentrate in the pleural space, has been shown to be an alternative biological marker for pleural TB diagnosis with pooled sensitivity and specificity estimates of 89% and 97%, respectively [5]. In high TB/HIV burden settings the performance was shown to be even better: sensitivity 97% and specificity 100% [6].

More recently the Xpert MTB/RIF assay, a fully automated quantitative real-time hemi-nested PCR, was introduced into high burden settings and is able to detect *M. tuberculosis* within 2 hours and also provide information about rifampicin susceptibility [7]. The assay has been validated using sputum samples and recently endorsed by the WHO as a rapid test for both smear-positive and smear-negative (paucibacillary) respiratory samples [8,9]. However, there are limited data about the Xpert MTB/RIF assay using pleural fluid [10-17] and thus the usefulness of this assay in the context of pleural TB remains unclear. Limitations of previously published work include the relatively small number of patients with pleural TB (usually quoted as part of a larger series of patients with EPTB), a paucity of biopsy-proven or culture positive samples as a gold standard, the lack of comparative analysis with other commonly used biomarkers, and a lack of attention to the technical factors that could impact Xpert MTB/RIF performance, including PCR inhibition, level of detection, and correlation with bacterial load. Furthermore, there are also limited data from high TB and HIV prevalence settings.

To address these knowledge gaps we prospectively evaluated the performance of the Xpert MTB/RIF assay, and other same-day diagnostic biomarkers, using pleural fluid obtained from patients with suspected pleural TB from Cape Town in South Africa.

## Methods

### Patient recruitment, characterization and routine laboratory tests

Consecutive patients with suspected pleural TB, including any symptoms including cough, fever, night sweats, loss of weight, haemoptysis and chest pain, and features consistent with a pleural effusion on chest x-ray, were prospectively recruited from Groote Schuur, Somerset and Victoria Hospitals in Cape Town, South Africa, over a three year period from October 2009 to September 2012. The University of Cape Town Human Research Ethics Committee approved the study, and all patients provided written informed consent for study participation and pleural biopsy.

Routine TB diagnostic work up (pleural fluid analysis, sputum for microscopy and culture, when available, and lymph node or other organ biopsy) was performed by the referring physician. Although not routine, a closed pleural biopsy using an Abrams needle was performed by a study physician trained in this procedure to improve patient categorization. All biopsies were performed after aspiration of pleural fluid. Patients were offered voluntary HIV testing. Pleural fluid samples were collected for routine biochemical and cytological analysis (protein, albumin, ADA, glucose, cell differential, cytology), concentrated fluorescence smear microscopy, and liquid culture for *M. tuberculosis* using the MGIT 960 (Becton Dickinson, Sparks, Maryland) with the remaining fluid used for Xpert MTB/RIF and IFN-γ analysis. Pleural biopsy samples were sent for histology and liquid culture. Adenosine deaminase activity in pleural fluid was determined by colorimetric technique by the National Health Laboratory Services, using the user-defined method on a Roche Cobas Integra (Roche Diagnostics Ltd, Switzerland). Pleural fluid ADA levels greater than 30 U/L, in keeping with local guidelines and clinical practice, were reported as suggestive of pleural TB [18,19].

Given the limitations of a single pleural fluid TB culture for confirming a diagnosis, patients were categorised as follows: *Definite*-TB: patients with at least one positive *M. tuberculosis* culture by liquid culture (in either pleural fluid, biopsy or sputum) and/or caseating granulomatous inflammation suggestive of TB on histological examination of pleural biopsy tissue, and with improvement on anti-TB treatment (all patients in this category received anti-TB treatment); *Probable*-TB: patients not meeting the criteria for definite-TB but with a clinical-radiological picture suggestive of TB and who were treated for TB with clinical response (all patients in this category received anti-TB treatment); *Non*-TB: patients for whom no microbiological or histological evidence of *M. tuberculosis* could be found, and/or for whom an alternative diagnosis was available. These patients at presentation and on follow-up did not receive anti-TB treatment.

All the laboratory staff performing the requested tests, including Xpert MTB/RIF and IFN-γ measurement, were blinded to all microbiological and clinical information.

### IFN-γ measurement

Interferon gamma (IFN-γ) concentrations were measured in pleural fluid supernatant in duplicate using the InterGam Ultrasensitive Rapid Immuno-suspension Assay (IRISA; Antrum Biotech, Cape Town, South Africa; limit of detection = 5 to 10 pg/ml). Pleural fluid supernatant was prepared by centrifuging pleural fluid at 3000×g for 15 min to remove any unwanted debris.

### Xpert MTB/RIF assay

A 1 ml aliquot of raw pleural fluid and a 1 ml aliquot of concentrated (centrifuged) pleural fluid from each patient was diluted with 2 ml of the Xpert MTB/RIF sample buffer. The 1 ml concentrated pleural fluid aliquot was prepared by centrifugation of a median (IQR) of 20 (10-20) ml pleural fluid at 3000×g for 15 min, with the supernatant discarded and the pellet made up to 1 ml with phosphate buffer solution. The pleural fluid and sample buffer solutions were then mixed vigorously and incubated at room temperature for 15 min, with further mixing halfway through the incubation. A 2 ml volume of the diluted samples was then transferred to an Xpert MTB/RIF cartridge and run on the GeneXpert (Cepheid, Dx System Version 4.0c) machine. The limit of detection was determined in duplicate by spiking 0, 50, 75, 100 and 150 H37Rv CFU to 1 ml aliquots of pleural fluid from subjects confirmed not to have TB, before dilution with sample buffer and subsequent Xpert MTB/RIF analysis. This experiment was repeated twice. Inhibition was evaluated by comparing the PCR cycle-threshold (C_T_) values of the internal positive control (lyophilized *Bacillus atrophaeus* subsp. *globigii* spores) from unconcentrated and concentrated samples.

### Statistical analysis

Categorical variables were compared using the χ^2^ test and continuous variables were compared using Student’s t-test where appropriate, with Mann-Whitney used for non-parametrically distributed continuous variables. Correlations were analyzed using the Spearman co-efficient for non-parametrically distributed variables. Diagnostic accuracy, including 95% confidence intervals, was assessed using sensitivity, specificity, positive predictive value (PPV), negative predictive value (NPV) (Open Epi, Version 2.3.1) and area under the receiver operator curve (AUROC) in definite-TB and non-TB groups (Graphpad Prism, Version 5.03).

## Results

**Figure 1:**
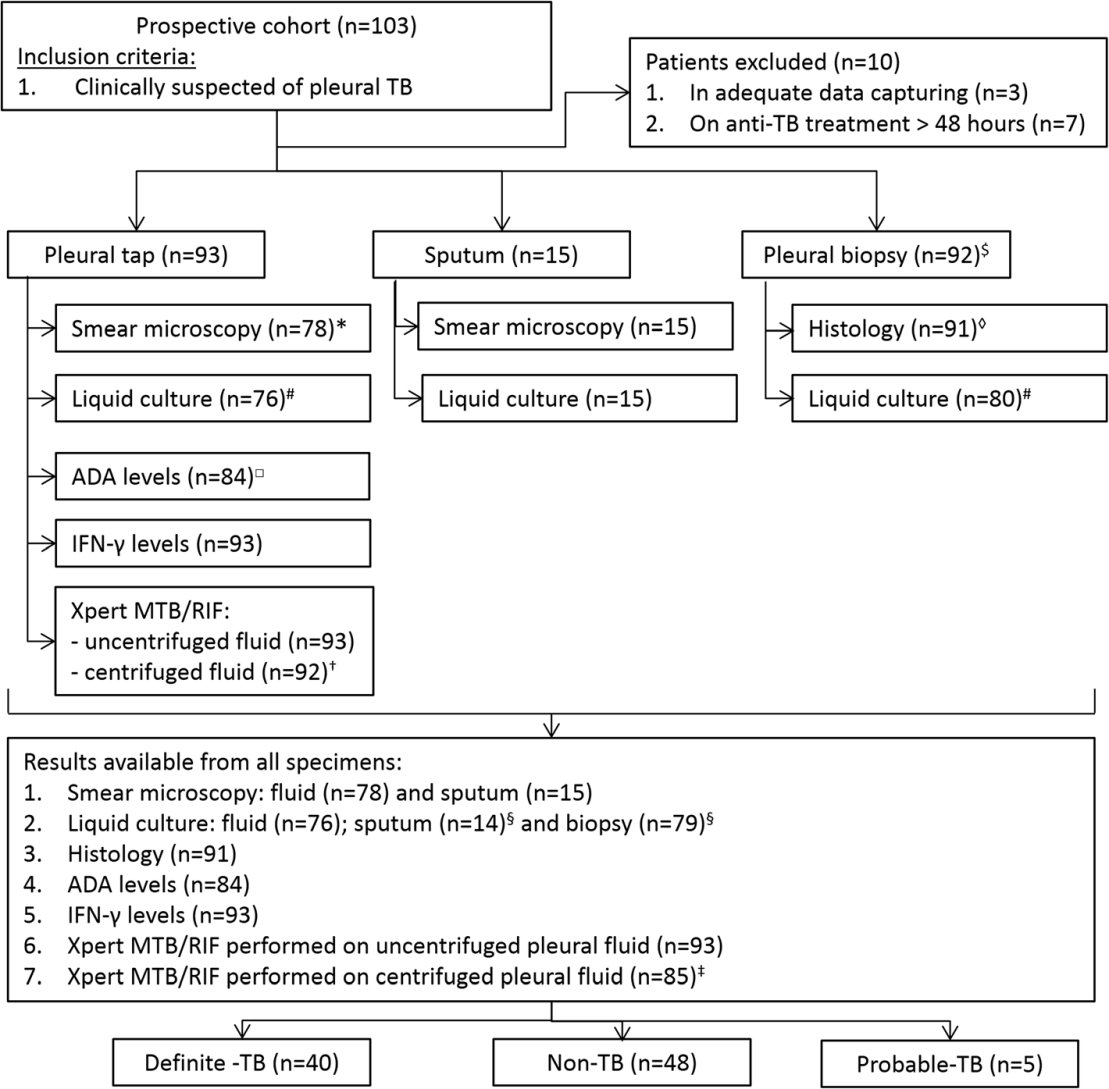
**Patient flow and test results available for final diagnoses. **^$^biopsy not performed (n = 1); *no fluid smear requested (n = 15); ^#^liquid culture not requested, pleural fluid (n = 17) and pleural biopsy (n = 12); ^§^indeterminate liquid culture: contamination (n = 1) and sample rejected (n = 1); ^◊^histology not requested (n = 1); ^□^ADA measurements not requested (n = 9); ^†^sample lost during processing (n = 1), ^‡^indeterminate Xpert MTB/RIFF result (n = 7).

A total of 103 patients with pleural effusion and suspected TB were enrolled. Ten patients were excluded from the final analysis: three had incomplete clinical data and seven were on anti-TB treatment for more than 48 hours prior to the samples being taken (see Figure 1 for patient flow and test results available).

### Clinical and demographic data

Forty subjects were confirmed to have definite-TB, 48 non-TB and 5 participants probable-TB (not meeting diagnostic criteria but receiving TB treatment). The subjects with non-TB effusions had a spectrum of malignant and non-malignant diagnoses including: lymphoma, adenocarcinoma, small cell carcinoma, and parapneumonic effusion. Of the 65 subjects consenting to HIV testing, 17% (11) were positive. Clinical and demographic data are summarized in Table 1.

**Table 1:** **Baseline characteristics of the definite tuberculosis** (**TB**) **and non**-**TB patients**

	**Definite-TB**	**Non-TB**	**p-value**
**Patients (n)**	40	48	
**Age (yrs)**	39 (29,55)	61 (54,69)	<0 · 0001^#^
**Sex**			
Male	24 (60%)	29 (60.4%)	
Female	16 (40%)	19 (39.6%)	
**Race**			
White	1 (2.5%)	5 (10 . 4%)	
Black	28 (70%)	9 (18.8%)	<0.0001*
Mixed	11 (27.5%)	34 (70.8%)	<0.0001*
**HIV Status**			
HIV-infected	8 (20%)	1 (2 . 0%)	<0.05*
HIV uninfected	22 (55%)	29 (60.4%)	
Refused testing	4 (10%)	3 (6.3%)	
Unknown	6 (15%)	15 (31.3%)	
**History of Previous TB**			
Yes	6 (15%)	5 (10.4%)	
No	31 (77 . 5%)	39 (81.3%)	
Unknown	3 (7.5%)	4 (8.3%)	
**BCG Status**			
Yes	20 (50%)	25 (52.1%)	
No	10 (25%)	5 (10.4%)	
Unknown	10 (25%)	18 (37.5%)	
**Current smoker**			
Yes	7 (17.5%)	18 (37.5%)	<0.05*
No	32 (80%)	26 (54.1%)	<0.05*
Unknown	1 (2.5%)	4 (8.3%)	

Data are presented as median (IQR) or n (%) or yrs (IQR). *BCG*: bacilli Calmette-Guerin. ^#^: unpaired t-test; *: Chi-squared test.

### Performance outcomes of IFN-γ

**Figure 2:**
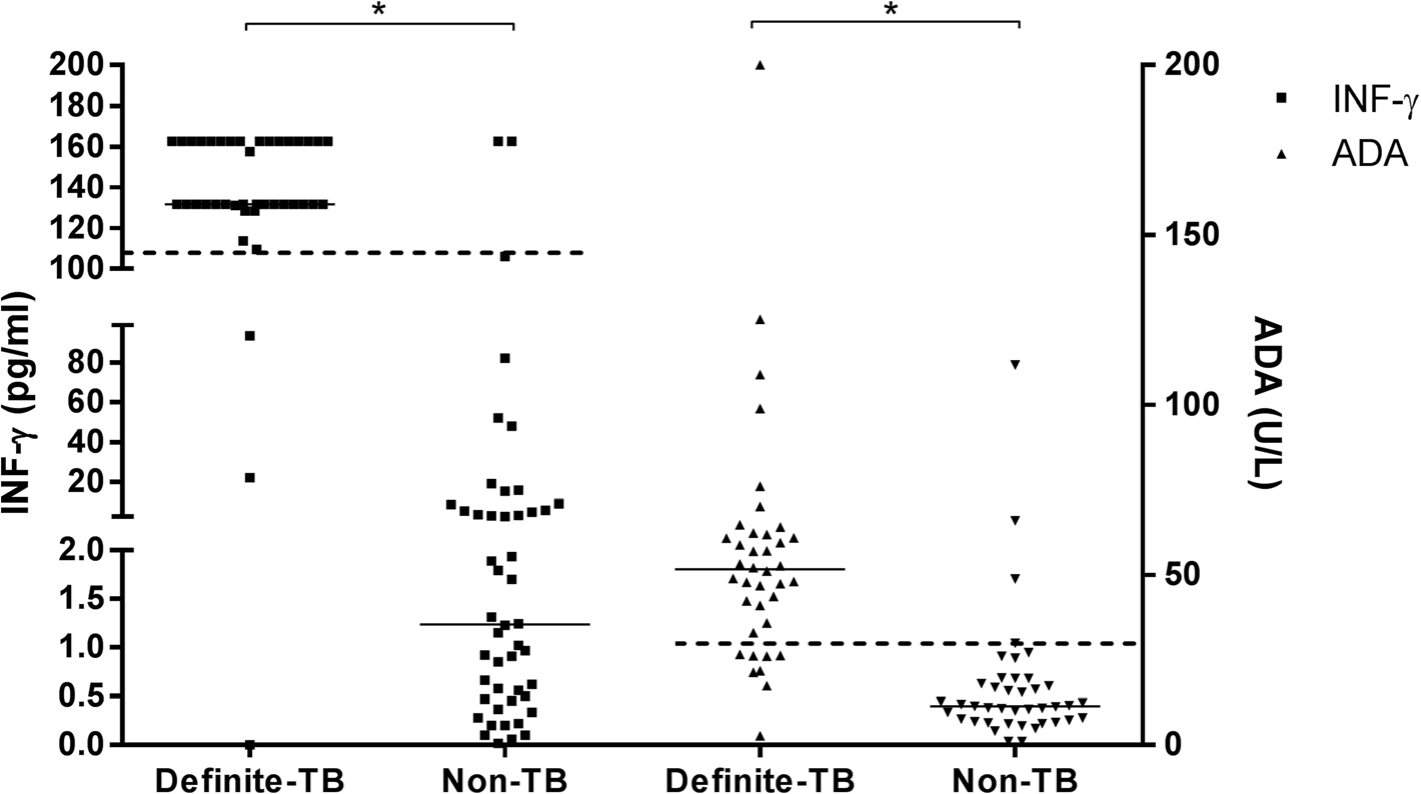
**Scatter plot of interferon gamma (IFN-γ) and adenosine deaminase (ADA) using pleural fluid from patients with TB and non-TB pleural effusions.** *: p < 0.0001 (Mann-Whitney). See Additional file 1: Table S6 in the online supplementary data for final diagnosis of non-TB patients with ADA and/or IFN-γ levels above cut points.

**Figure 3:**
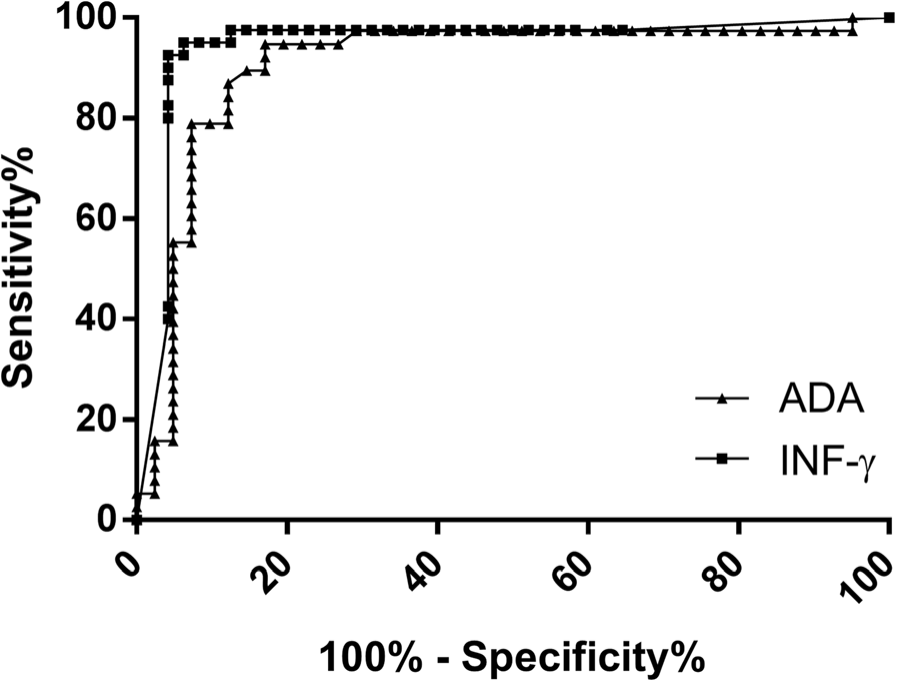
Area under the receiver operator characteristic (ROC) curves for interferon gamma (IFN-γ) and adenosine deaminase (ADA) were 0.94 and 0.91, respectively.

IFN-γ levels were evaluated in 93 patients (40 definite-TB, 5 probable-TB and 48 non-TB). Median (IQR) IFN-γ levels were approximately 100 times higher in definite-TB compared to non-TB effusions: 131.8 (131.8-162.7) versus 0.57 (0.0-5.71) pg/ml, p < 0.0001 (Figure 2). A receiver operating characteristic (ROC) curve derived rule-in cut point of 107.7 pg/ml was used to determine the sensitivity and specificity (95% CI) (Figure 3). Table 2 compares the diagnostic accuracy of the IFN-γ with other same-day diagnostics in definite-TB versus non-TB groups. IFN-γ sensitivity was unaffected by the inclusion of probable- with definite-TB patients (see Additional file 1: Table S1 in the online supplementary data), and diagnostic accuracy was not significantly different in HIV-infected compared to uninfected patients (see Additional file 1: Table S2 in the online supplementary data).

**Table 2:** **Per patient diagnostic accuracy of Xpert MTB**/**RIF**, **IFN**-**γ**, **and ADA for the diagnosis pleural tuberculosis**

**Assay**	**Definite-TB vs. Non TB**					
	**Sensitivity% (95% CI)**	**Specificity% (95% CI)**	**PPV% (95% CI)**	**NPV% (95% CI)**	**+LR (95% CI)**	-**LR (95% CI)**
Xpert MTB/RIF	22.5% *^#□$£^ (12.4, 37.6) 9/40	98% (89.2, 99.7) 47/48	90% (59.6, 98.3) 9/10	60.3% ^†◊●▪^ (49.2, 70.4) 48/78	10.8 (1.43, 81.65)	0.79^¢°¤§¿^ (0.67, 0.94)
IFN-γ	92.5%*^O^ (80.2, 97.5) 37/40	95.9% (86.1, 98.9) 46/48	94.9% (83.2, 98.6) 37/39	93.9%^†∞^ (83.5, 97.9) 46/49	22.20 (5.70, 86.46)	0.08^¢ð^ (0.03, 0.23)
ADA (Clinical cut point)	79% ^∆#×^ (63.7, 89) 30/38	92.7% (80.6, 97.5) 38/41	91% (73.4, 95.4) 30/33	82.7% ^‡◊^ (69.3, 91.0) 38/46	10.79 (3.59, 32.47)	0.23^°^ (0.12, 0.42)
ADA (Rule-in cut point)	55.3% ^□O×€¥^ (39.8, 69.9) 21/38	95.2% (83.9, 98.7) 39/41	91.4% (73.3, 97.6) 21/23	69 · 7% ^∞⌂^ (56.7, 80.1) 39/56	11.33 (2.85, 45.1)	0.47^¤ðþ^ (0.33, 0.67)
Xpert MTB/RIF followed by IFN-γ if MTB/RIF-negative	92.5%^$€^ (80.2, 97.5) 37/40	93.8% (83.2, 97.9) 45/48	92.5% (80.2, 97.5) 37/40	93.8%^●⌂^ (83.2, 97.9) 45/48	14.80 (4.93, 44.43)	0.08^§þ^ (0.03, 0.24)
Xpert MTB/RIF followed by ADA (clinical cut-point) if MTB/RIF-negative	81.6%^£¥^ (66.6, 90.8) 31/38	92.7% (80.6, 97.5) 38/41	91.2% (77.1, 97) 31/34	84.5%^▪^ (71.3, 92.3) 38/45	11.15 (3.70, 33.49)	0.20^¿^ (0.10, 0.39)
p-value	*; ^#^; ^$^ and ^£^: p < 0.0001 ^O^; ^□^; ^×^; ^€^ and ^¥^: p < 0.05			^†^ and ^●^: P < 0.0001 ^◊^; ^∞^; ▪ and ^⌂^: p < 0.05		^¢^; ^°^; ^¤^; ^§^; ^¿^; ^ð^ and ^þ^: non overlapping CI

A positive *MTB* (*Mycobacterium tuberculosis*) fluid culture and/or positive *MTB* biopsy culture and/or histology in keeping with *MTB* infection used as a reference. *PPV*: positive predictive value, *NPV*: negative predictive value, +*LR*: positive likelihood ratio, -*LR*: negative likelihood ratio, *CI*: confidence interval, *IFN*-γ: interferon gamma, *ADA*: adenosine deaminase. *IFN*- γ cut point of 107.7 pg/ml determined by ROC. ADA clinical cut point of 30 U/L is used for clinical decision-making at Groote Schuur hospital. Rule-in ADA cut point of 48.85U/l determined by ROC.

Superscript symbols: *; ^#^; ^$^; ^£^: ^O^; ^□^; ^×^; ^€^; ^¥^; ^†^; ^●^; ^◊^; ^∞^; ▪; ^⌂^; ^¢^; ^°^; ^¤^; ^§^; ^¿^; ^ð^ and ^þ^were used to indicate which groups were being compared for statistical analysis.

### Performance outcomes of pleural fluid ADA

Adenosine deaminase levels were measured in 84 patients. Median (IQR) ADA levels were 5 times higher in definite-TB compared to non-TB effusions: 51.7 (35.2-62.1) versus 11.5 (7.1-18.8) U/L, p < 0.0001. Using a clinical cut point of >30 U/L (South African national standard [18]), the sensitivity (95%CI), specificity, NPV and PPV were 79.0% (63.7-89.0), 92.7% (80.6-97.5), 82.7% (69.3-91.0) and 91.0% (73.4-95.4), respectively (Table 2). Relevant values using the rule-in cut-point (at least 95% specificity) are shown in Table 2. The ROC and scatter plot of ADA are shown in Figures 2 and 3.

Grouping the probable TB with the definite-TB group reduced the sensitivity to 74.5% but the specificity remained unchanged, when using the clinically used cut point of >30 U/L. A similar trend occurred when using the rule-in cut point of 48.85 U/L (see Additional file 1: Table S1 in the online supplementary data). HIV status had no significant impact on the ADA diagnostic accuracy (see Additional file 1: Table S3 in the online supplementary data).

### Performance outcome for Xpert MTB/RIF assay including inhibition and detection threshold

Xpert MTB/RIF was performed on 93 participants. Xpert MTB/RIF detected 9 out 40 subjects with definite-TB. The sensitivity (95%CI) and specificity using 1 ml of unprocessed pleural fluid was 22.5% (12.4, 37.6) and 98.0% (89.2, 99.7) respectively. Xpert MTB/RIF sensitivity and specificity did not improve when a concentrated (centrifuged) pleural fluid samples was used (p = 0.90, Table 3). However, the number of indeterminate results generated in concentrated compared to unprocessed pleural fluid samples was higher 7/92 vs. 0/93 (p < 0.05). Table 2 compares the diagnostic accuracy of Xpert MTB/RIF and other same-day diagnostics. Of note, Xpert MTB/RIF pleural fluid testing detected one rifampicin-resistant result that was confirmed by liquid culture to be a true-positive MDR-TB case.

**Table 3:** **Per patient diagnostic accuracy of Xpert MTB**/**RIF assay using centrifuged and uncentrifuged pleural fluid to diagnose pleural tuberculosis**

**Assay**	**Definite-TB vs. Non-TB**			
	**Sensitivity% (95% CI)**	**Specificity% (95% CI)**	**PPV% (95% CI)**	**NPV% (95% CI)**
Pleural fluid (uncentrifuged)	22.5% (12.4, 37.6) 9/40	98% (89.2, 99.7) 48/48	90% (59.6, 98.3) 9/10	60.3% (49.2, 70.4) 48/78
Centrifuged pellet	23.7% (13.0, 39.3) 9/38	100% (91.7, 100) 42/42	100% (70.1, 100) 9/9	59.2% (48.2, 70.3) 42/71
p-value	ns*	ns*	ns*	ns*

A median volume of 20 ml of pleural fluid was centrifuged at 3000 × g for 15 min with the pellet resuspended in sterile PBS, followed by Xpert *MTB/RIF*. A positive *MTB* (*Mycobacterium tuberculosis*) fluid culture and/or positive MTB biopsy culture and/or histology in keeping with MTB infection used as a reference. *PPV*: positive predictive value, *NPV*: negative predictive value, *CI*: confidence interval, *: Chi squared.

**Figure 4:**
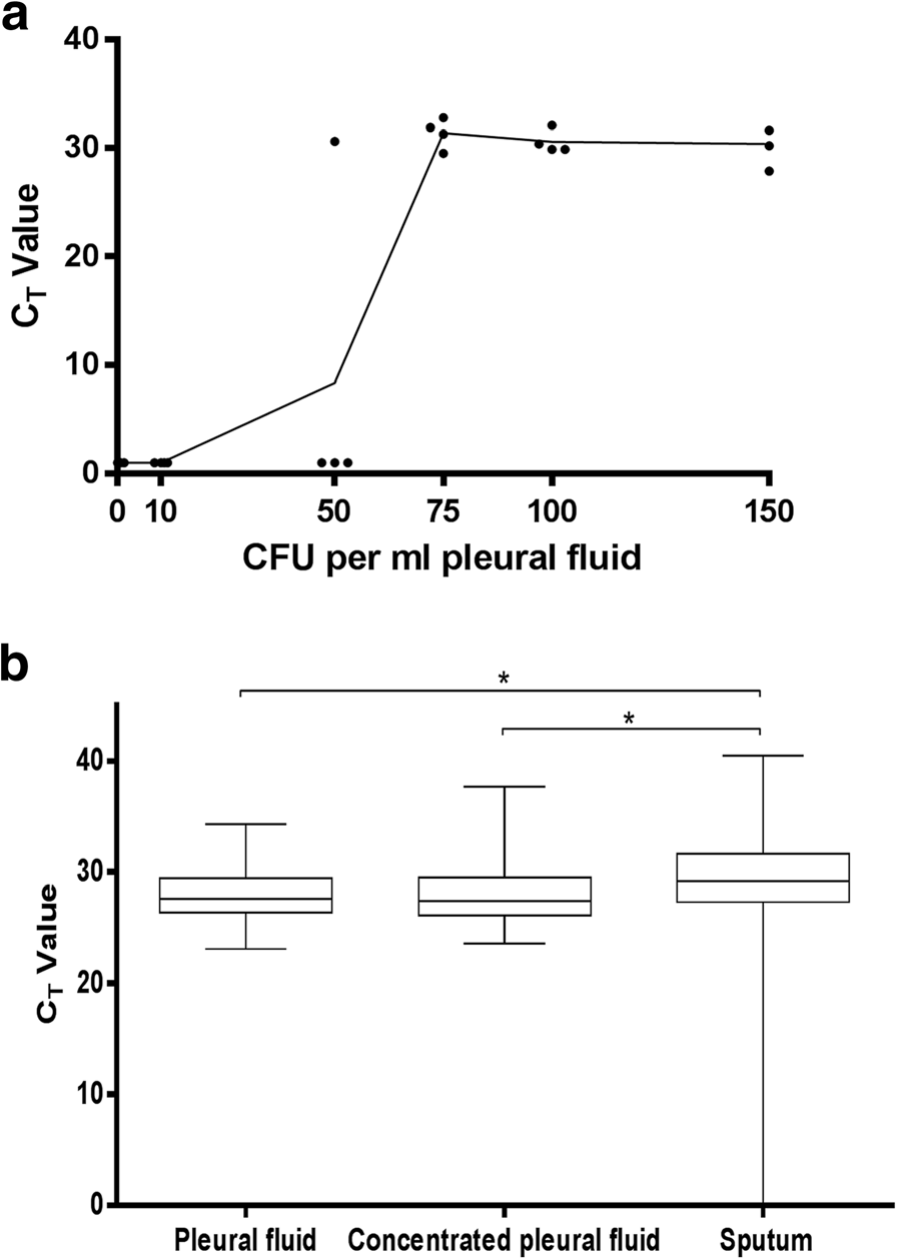
**The determination of the: a) limit of detection of Xpert MTB/RIF and b) analysis of possible inhibition of Xpert MTB/RIF internal probe in pleural fluid samples. a)** To determine the limit of detection, known amounts of colony forming units of stock H37Rv were added to 1 ml aliquots of pleural fluid in duplicate for two repeat experiments. **b)** CT values for Xpert MTB/RIF internal probe are represented as box and whiskers (min and max) for pleural fluid, concentrated pleural fluid (20 ml pellet resuspended in 1 ml) and sputum. *: p < 0.0001 (Mann-Whitney).

Spiking raw pleural fluid demonstrated that the Xpert MTB/RIF assay was able to reliably detect ≥75 CFU per millilitre of pleural fluid (Figure 4a). No significant difference was seen between unprocessed and concentrated pleural fluid internal probe amplifications with median C_T_ values of (95% CI) of 27.6 (27.5, 28.5) and 27.4 (27.6, 28.9), respectively. The median time-to-positivity (TTP) (IQR) for pleural fluid and pleural biopsy culture were 28.0 days (23.3-31.1) and 19.0 days (17.7-24.6), respectively (data not shown). No correlation was observed between C_T_ values and TTP from pleural fluid liquid culture, however, there was a correlation (Spearman r = 0.93, p = 0.02) between C_T_ values and the TTP from pleural biopsy culture.

In contrast to the findings with ADA and IFN-γ, when subjects were stratified by HIV status, Xpert MTB/RIF had a higher sensitivity in HIV infected versus uninfected persons [50% (21.6-78.5) vs. 9.1% (2.6-27.9), p = 0.031 (see Additional file 1: Table S4 in the online supplementary data). A similar trend was seen when comparing pleural fluid and biopsy culture as reference standard (see Additional file 1: Table S5 in the online supplementary data).

Xpert MTB/RIF in combination with ADA or IFN-γ did not significantly improve the diagnostic accuracy when compared to ADA or IFN-γ alone (Table 2).

## Discussion

Given that a reliable same-day diagnostic tool for pleural TB is lacking, we prospectively evaluated and compared the use of ADA, IFN-γ and Xpert MTB/RIF using pleural fluid from patients with suspected TB. Our key findings were (i) Xpert MTB/RIF had poor sensitivity and this was neither the result of a sub-optimal level of detection compared to other types of biological samples nor increased PCR inhibition, (ii) Xpert MTB/RIF sensitivity was not improved by concentrating larger volumes of pleural fluid, (iii) ADA and IFN-γ are good rule-in tests for TB pleural effusions but the higher sensitivity of IFN-γ would be preferable over ADA for rule-in, particularly in low-burden settings where a high INF-γ level is unlikely to be due to cancer, (iv) the excellent rapid rule-out value of IFN-γ, compared to ADA, is of particular clinical usefulness in high burden settings as it could be used to prompt a search for an alternative diagnosis and hence medical or surgical biopsy. The sheer burden of disease and resource constraints, including lack of skilled operators, in high burden settings means that a routine biopsy-based approach cannot be undertaken. Moreover, the majority of patients suspected with pleural TB were unable to produce sputum.

The Xpert MTB/RIF assay, which can simultaneously detect the presence of *M. tuberculosis* and rifampicin resistance in sputum specimens, has shown great promise in the rapid diagnosis of TB, with an average sensitivity and specificity of 90.4% and 98.4%, respectively [8]. It has also improved the rapid diagnosis of pulmonary TB (sensitivity of ~68%) in smear-negative patients and recently been endorsed by the Scientific and Technical Advisory Board of the WHO for use in paucibacillary samples [9,20]. However, there are hardly any data about Xpert performance in pleural fluid. Published studies have generally been performed in low burden settings as a laboratory exercise reporting yields in samples from extra-pulmonary sites, and only reported on a handful of patients with TB pleural effusion [10,12-16]. This may explain the high sensitivity reported in these studies (range 58% to 100%) and high specificities (87% to 100%) [10-16]. Recently Friedrich and co-workers in a selected cohort found that 20 out of 25 patients had confirmed TB and that Xpert sensitivity was 25% [11]. We have confirmed these findings in a larger unselected cohort and further interrogated the technical performance of the assay. The level of detection was ≥75 colony forming units per ml, and there was no evidence of PCR inhibition using the internal positive control. Centrifugation of pleural fluid made little difference. The time to positivity data (median > 28 days) confirms the low organism load within the pleural space compared to pleural tissue. Indeed, there was a trend to shorter time to positivity in liquid culture from samples that were culture positive/Xpert positive compared to those that were culture positive/Xpert negative (data not shown). Collectively, these data confirm the notion that pleural TB is a paucibaciliary disease and thus concentration/ centrifugation of at least 20 ml of pleural fluid makes little difference to diagnostic yield. Xpert performed better in HIV-infected individuals, which may reflect a higher organism load in the pleural fluid [21].

In high burden settings such as South Africa, a high ADA level is frequently used to guide initiation of anti-TB therapy [4,5]. In this study, although ADA levels were 5 times higher in TB patients than in non-TB patients, using the laboratory accepted cut-point in Cape Town (30 U/L) roughly 20% of TB patients would have been missed and 1 in 10 incorrectly started on anti-TB therapy. Despite the inability of ADA to definitively confirm *M. tuberculosis*, it is a low cost and relatively rapid (same day) assay and has a high PPV when disease prevalence is high [22]. In low prevalence settings, however, PPV is too low to be clinically useful. Indeed, a recent meta-analysis of 63 studies including 2796 pleural TB patients and 5297 non-TB patients reported the sensitivity of ADA to be 92% and specificity 90% at a cut-point of 41.9 U/L (median cut-point in the pooled studies, which each used a different cut-point) [4]. Thus, 1 in 10 patients would be over diagnosed with TB. The specificity of ADA may be improved if the proportion of lymphocytes is taken into account as was recently shown [22]. However, this is not universally helpful as about ~25% of TB pleural effusions , may be neutrophil predominant [23].

Interferon gamma, similarly, was significantly higher in TB (100 fold) compared to non-TB patients. Using an ROC curve-determined cut-point of 107.7 pg/ml, only 3 definite-TB patients were missed. By contrast, Xpert sensitivity, like pleural fluid culture (~40%) [2], was poor and therefore additional tools are required for optimal diagnosis when a non-biopsy approach is used. Thus, a ‘one size fits all’ approach with Xpert is inappropriate. Although closed pleural biopsy has a high diagnostic yield it is often unavailable in resource-poor settings, and even when available the large numbers of cases preclude routine biopsy in district general hospitals. In this study we have confirmed our previous findings, and those of others, that IFN-γ is both a highly accurate rule-in and rule-out diagnostic test for pleural TB [6,24-26], however it must be accepted that the 107.7 pg/ml cut point was generated using the current cohort and further prospective testing is required to confirm the high sensitivity and specificity. Although it can be easily measured by a commercially available ELISA-kit, it is not routinely performed due to the high cost and the kits only being available in a 96 well format, which lead to a considerable wastage of unused wells [27]. Interestingly, of the only two non-TB patients that had elevated IFN-γ, one patient had an empyema, which is known to cause elevated IFN-γ and is a contra-indication to using the test, and the other patient was lost to follow-up and thus the true TB status was unclear. A previous study from our group demonstrated a similar accuracy (sensitivity 97% specificity 98%) [6]. An obvious drawback is the lack of susceptibility data, but in resource-poor settings culture confirmation with susceptibility can follow if there is a poor response to treatment. In any event a culture isolate or positive NAAT would be required to determine susceptibility- the yield of both are low in pleural TB. It would have been interesting to perform Xpert on the pleural tissue biopsies but technically this was not feasible due to the limited amount of tissue and potential for tube blockage in the machine when solid material is used. However this may not have improved the diagnostic accuracy, in a recent study, Xpert was unable to detect any TB cases and more indeterminate results occurred when performed on finely ground pleural tissue [17].

Affordability and cost effectiveness remains an important consideration in resource poor TB endemic countries. A comprehensive cost effectiveness analysis was beyond the scope of this paper, and we were unable to perform a simple cost analysis given the lack of a clinically validated commercially available unstimulated interferon gamma assay. Although the GeneXpert MTB/RIF assay is now being rolled out in many TB endemic countries [28], as we have demonstrated, sensitivity is largely sub-optimal [6,29]. Although ADA is widely available, specificity may also be also sub-optimal, as we and others have previously demonstrated. Nevertheless, it remains a widely available relatively low cost test. Diagnosing drug-resistant pleural TB also merits cost consideration. However, the GeneXpert MTB/RIF assay has a poor sensitivity in this context and thus whatever diagnostic modality is used (unstimulated interferon gamma, ADA, or GeneXpert MTB/RIF) pleural tissue or fluid culture is still required for susceptibility testing. There is an urgent need to make available a commercially and clinically validated, relatively rapid, single patient use assay for the measurement of unstimulated interferon gamma levels in pleural fluid and other forms of EPTB.

There are several limitations of our study. There were a low proportion of HIV-infected patients and several patients with unknown HIV status. However, HIV prevalence rates in TB patients in the Western Cape Province of SA are known to be lower than in the rest of the country [30], and patients often refuse testing. We only centrifuged 20 ml of fluid. However, we were limited by available sample, particularly when effusions were loculated. Moreover, a recent paper has showed that increasing the pleural fluid volume to 100 ml does not improve culture yields, despite improving the time to positivity [21]. The conclusions drawn here apply to high TB and HIV burden settings and, for the sake of external validity, require confirmation in other settings. However, given that HIV increases the concentration of organisms in pleural fluid and that the upper limit of the Xpert sensitivity (95% CI) was 37% suggests that Xpert is unlikely to perform well in any setting when using pleural fluid. We did not evaluate the potential impact on morbidity and length of hospital stay of ADA, IFN-γ and Xpert compared to empiric treatment based on laboratory analysis alone (lymphocyte predominance), however, this would have require a randomized design and up to ~25% of TB effusions are known to be neutrophil pre-dominant [23]. The confidence intervals of the sensitivity estimates for interferon gamma and the GeneXpert MTB/ RIF assay are not ideal and therefore our findings should be confirmed using larger sample numbers and from different parts of the world.

## Conclusion

The clinical usefulness of Xpert-MTB/RIF to diagnose pleural TB is limited by its poor sensitivity. By contrast, IFN-γ is an excellent rule-in test and, compared to ADA, has significantly better sensitivity and rule-out value in a high HIV prevalence setting. The high NPV of IFN-γ, compared to ADA, is particularly useful to clinicians as it prompts further work-up and tissue biopsy in patients who are unlikely to have TB, however further prospective testing is required.

## Competing interests

KD, UG and GT have performed consultancy work for Antrum Biotech (Pty) Ltd, a UCT co-owned spin-off company, and kits for the study were donated by the company. However, Antrum Biotech played no role in study design, data analysis or its publication. Similarly, KD has been the recipient of grant funding from FIND Diagnostics, who also donated Xpert MTB/RIF cartridges to carry out the study but FIND played no role in study design, data analysis or its publication.

## Authors’ contributions

Concept and design, KD, RM, GT, JP. Laboratory experiments, RM, AB. Analysis and interpretation, RM, RvZS, JP, GC, GS, GN, BA, HK. Drafting the manuscript for important intellectual content, KD, RM, RvZS, GT, JP, UG. All authors read and approved the final manuscript.

## Pre-publication history

The pre-publication history for this paper can be accessed here:

http://www.biomedcentral.com/1471-2466/14/58/prepub

## Supplementary Material

Additional file 1: Table S1. Per patient diagnostic accuracy of Xpert MTB/RIF, IFN-γ, and ADA for the diagnosis pleural tuberculosis. **Table S2.** Per patient diagnostic accuracy of IFN-γ for the diagnosis of pleural tuberculosis, stratified by HIV status. **Table S3.** Per patient diagnostic accuracy using ADA for the diagnosis of pleural tuberculosis, stratified by HIV status. **Table S4.** Per patient diagnostic accuracy of the Xpert MTB/RIF assay, stratified by HIV status. **Table S5.** Per sample diagnostic accuracy of the XpertMTB/RIF assay for the diagnosis of pleural tuberculosis, using either fluid or biopsy culture as reference standard. Stratified by HIV status. **Table S6.** Non-TB patients with ADA and/or INF-γ levels above specified cut points. Click here for file
