# Supplementary material for: Comparison of same day diagnostic tools including Gene Xpert and unstimulated IFN-γ for the evaluation of pleural tuberculosis: a prospective cohort study
Source: BMC Pulm Med. 2014 Apr 8;14:58. doi: 10.1186/1471-2466-14-58 (PMC4108018; doi:10.1186/1471-2466-14-58)
Supplement: Additional file 1: Table S1. — Per patient diagnostic accuracy of Xpert MTB/RIF, IFN-γ, and ADA for the diagnosis pleural tuberculosis. Table S2. Per patient diagnostic accuracy of IFN-γ for the diagnosis of pleural tuberculosis, stratified by HIV status. Table S3. Per patient diagnostic accuracy using ADA for the diagnosis of pleural tuberculosis, stratified by HIV status. Table S4. Per patient diagnostic accuracy of the Xpert MTB/RIF assay, stratified by HIV status. Table S5. Per sample diagnostic accuracy of the XpertMTB/RIF assay for the diagnosis of pleural tuberculosis, using either fluid or biopsy culture as reference standard. Stratified by HIV status. Table S6. Non-TB patients with ADA and/or INF-γ levels above specified cut points. [file 1471-2466-14-58-S1.docx]

Table E1: Per patient diagnostic accuracy of Xpert MTB/RIF, IFN-γ, and ADA for the diagnosis pleural tuberculosis.

| **Assay** | **Definite-TB and probable-TB combined vs. Non TB** | | | | | |
| --- | --- | --- | --- | --- | --- | --- |
|  | Sensitivity% (95% CI) | Specificity% (95% CI) | PPV%  (95% CI) | NPV%  (95% CI) | +LR | -LR |
| Xpert MTB/RIF | 24.5% *^#□$£^ (14.3, 39.5)  11/45 | 98% (89.2, 99.7)  47/48 | 81.9% (52.4, 94.9)  9/11 | 58.1% ^†◊●▪^ (47.2, 68.2)  47/81 | 11.73  (1.58, 87.25) | 0.77^¢º¤§^  (0.65, 0.92) |
| IFN-γ | 92.5%*^∆O^ (80.2, 97.5)  37/40 | 95.9% (86.1, 98.9)  46/48 | 100% (90.6, 100)  37/37 | 93.9%^†^^‡∞^ (83.5, 97.9)  46/49 | 22.20  (5.70, 86.46) | 0.08^¢^  (0.03, 0.23) |
| ADA  (Clinical cut point) | 74.5% ^∆#×¢^  (59.8, 85.1)  32/43 | 92.7% (80.6, 97.5)  38/41 | 93.8% (79.9, 98.3)  30/32 | 77.6% ^‡◊^^ (64.2, 87)  38/49 | 10.17  (3.37, 30.66) | 0.28^º^  (0.17, 0.46) |
| ADA  (Rule-in cut point) | 53.5% ^□O×€¥^ (39, 67.5)  23/43 | 95.2% (83.9, 98.7)  39/41 | 91.4% (73.3, 97.6)  21/23 | 66.2% ^∞⌂^ (53.4, 76.9)  39/59 | 10.97  (2.76, 43.59) | 0.49^Þ^  (0.35, 0.68) |
| Xpert MTB/RIF followed by IFN-γ if MTB/RIF-negative | 92.9%^$€¢^ (81, 97.6)  39/42 | 93.8% (83.2, 97.9)  45/48 | 92.9% (81, 97.6)  39/42 | 93.8%^●⌂^^ (83.2, 97.9)  45/48 | 16.17  (5.38, 48.58) | 0.05^¤Þ^  (0.01, 0.20) |
| Xpert MTB/RIF followed by ADA (clinical cut-point) if MTB/RIF-negative | 79.1%^£¥^ (64.8, 88.6)  34/43 | 92.7% (80.6, 97.5)  38/41 | 91.9% (78.7, 97.3)  34/37 | 80.9%^▪^ (67.5, 89.6)  38/47 | 10.81  (3.60, 32.47) | 0.23^§^  (0.13, 0.41) |
| p-value | *; ^#^; ^$^; ^£^and ^€^: p<0.0001  ^O^; ^□^; ^×^; ^¥^ and ^¢^: p<0.05 |  |  | ^†^ and ^●^: P<0.0001  ^◊^; ^‡^; ^∞^; ^▪^; ^⌂^and ^^^: p<0.05 |  | ^¢^; ^º^; ^¤^;^§^ and ^Þ^: non overlapping CI |
| **Assay** | **Definite-TB vs. Non-TB and probable TB combined** | | | | | |
|  | Sensitivity% (95% CI) | Specificity%  (95% CI) | PPV% (95% CI) | NPV% (95% CI) | +LR | -LR |
| Xpert MTB/RIF | 22.5% *^#□$£^ (12.4, 37.6)  9/40 | 94.4% (84.7, 98.1)  50/53 | 75% (46.8, 91.2)  9/12 | 61.8% ^†◊●▪^ (50.9, 71.6)  50/81 | 3.98  (1.15, 13.74) | 0.82^¢º¤§^  (0.69, 0.98) |
| IFN-γ | 92.5%*^O^ (80.2, 97.5)  37/40 | 95.9% (86.1, 98.9)  46/48 | 94.9% (83.2, 98.6)  37/39 | 93.9%^†∞^ (83.5, 97.9)  46/49 | 22.20  (5.70, 86.46) | 0.08^¢Þ^  (0.03, 0.23) |
| ADA  (Clinical cut point) | 79% ^#×^ (63.7, 89)  30/38 | 89·2% (77, 95.3)  41/46 | 85·8% (70.7, 93.8)  30/35 | 83·7% ^◊^ (71, 91.5)  41/49 | 7.26  (3.12, 16.89) | 0.24^º^  (0.13, 0.44) |
| ADA  (Rule-in cut point) | 55.3% ^□O×€¥^ (39.8, 69.9)  21/38 | 91.4% (79.7, 96.6)  42/46 | 84% (65.4, 93.6)  21/25 | 71.2% ^∞⌂^ (58.7, 81.2)  42/59 | 6.36  (2.39, 16.92) | 0.49^Þð^  (0.34, 0.71) |
| Xpert MTB/RIF followed by IFN-γ if MTB/RIF-negative | 92.5%^$€^ (80.2, 97.5)  37/40 | 90% (78.7, 95.7)  45/50 | 88.1% (75, 94.9)  37/42 | 93.8%^●⌂^ (83.2, 97.9)  45/48 | 9.25  (4.01, 21.35) | 0.08^¤ð^  (0.03, 0.25) |
| Xpert MTB/RIF followed by ADA (clinical cut-point) if MTB/RIF-negative | 81.6%^£¥^ (66.6, 90.8)  31/38 | 87% (74.4, 93.9)  40/46 | 83.8% (68.9, 92.4)  31/37 | 85.2%^▪^ (72.4, 92.6)  40/47 | 6.25  (2.92, 13.39) | 0.21^§^  (0.11, 0.42) |
| p-value | *; ^#^; ^$^ and ^£^: p<0.0001  ^O^; ^□^; ^×^; ^€^ and ^¥^: p<0.05 |  |  | ^†^ and ^●^ : P<0.0001  ^◊^; ^∞^;  ^▪^ and ^⌂^: p<0.05 |  | ^¢^; ^º^; ^¤^;^§^; ^Þ^ and ^ð^: non overlapping CI |

A positive MTB (*Mycobacterium tuberculosis*) fluid culture and/or positive MTB biopsy culture and/or histology in keeping with MTB infection used as a reference. PPV: positive predictive value, NPV: negative predictive value, +LR: positive likelihood ratio, -LR: negative likelihood ratio, CI: confidence interval, IFN-γ: interferon gamma, ADA: adenosine deaminase. IFN-γ cut point of 107.7 pg/ml determined by ROC. ADA clinical cut point of 30 U/L is used for clinical decision-making at Groote Schuur hospital. Rule-in ADA cut point of 48.85U/l determined by ROC.

Table E2: Per patient diagnostic accuracy of IFN-γ for the diagnosis of pleural tuberculosis, stratified by HIV status.

| Patients | Definite-TB vs. Non-TB | | | |
| --- | --- | --- | --- | --- |
|  | Sensitivity%  (95% CI) | Specificity%  (95% CI) | PPV%  (95% CI) | NPV%  (95% CI) |
| All patients | 92.5% (80.2, 97.5)  37/40 | 95.9% (86.1, 98.9)  46/48 | 94.9% (83.2, 98.6)  37/39 | 93.9% (83.5, 97.9)  46/49 |
| HIV un-infected | 91% (72.2, 97.5)  20/22 | 96.6% (82.9, 99.4)  28/29 | 95.3% (77.4, 99.2)  20/21 | 93.4% (78.7, 98.2)  28/30 |
| HIV infected | 87.5% (53, 97.8)  7/8 | 100% (20.7, 100)  1/1 | 100% (64.6, 100)  7/7 | 50% (9.5, 90.6)  1/2 |

IFN-γ: interferon gamma, PPV: positive predictive value, NPV: negative predictive value, CI: confidence interval.

Table E3: Per patient diagnostic accuracy using ADA for the diagnosis of pleural tuberculosis, stratified by HIV status.

| Patients | Definite-TB vs. Non-TB | | | |
| --- | --- | --- | --- | --- |
|  | Sensitivity%  (95% CI) | Specificity%  (95% CI) | PPV%  (95% CI) | NPV%  (95% CI) |
| All patients | 79% (63.7, 89)  30/38 | 92.7% (80.6, 97.5)  38/41 | 91% (76.5, 96.9)  30/33 | 82.7% (69.3, 91)  38/46 |
| HIV un-infected | 65% (43.3, 81.9)  13/20 | 92.4% (75.9, 97.9)  24/26 | 86.7% (62.2, 96.3)  13/15 | 77.5% (60.2, 88.7)  24/31 |
| HIV infected | 100% (67.6, 100)  8/8 | 100% (20.7, 100)  1/1 | 100% (67.6, 100)  8/8 | 100% (20.7, 100)  1/1 |

ADA: adenosine deaminase, PPV: positive predictive value, NPV: negative predictive value, CI: confidence interval. ADA clinical cut point of 30 U/l is used for clinical decision-making at Groote Schuur hospital.

Table E4: Per patient diagnostic accuracy of the Xpert MTB/RIF assay, stratified by HIV status.

| Sample type | Patients | Definite-TB vs. Non-TB | | | |
| --- | --- | --- | --- | --- | --- |
|  |  | Sensitivity%  (95% CI) | Specificity%  (95% CI) | PPV%  (95% CI) | NPV%  (95% CI) |
| Fluid | All patients | 22.5% (12.4, 37.6)  9/40 | 98% (89.2, 99.7)  47/48 | 90% (59.6, 98.3)  9/10 | 60.3% (49.2, 70.4)  47/78 |
|  | HIV un-infected | 9.1% * (2.6, 27.9)  2/22 | 96.6% (82.9, 99.4)  28/29 | 66.7% (20.8, 93.9)  2/3 | 58.4% (44.3, 71.2)  29/49 |
|  | HIV infected | 50% * (21.6, 78.5)  4/8 | 100% (20.7, 100)  1/1 | 100% (51.1, 100)  4/4 | 20% (3.7, 62.5)  1/5 |

A positive MTB (*Mycobacterium tuberculosis*) fluid culture and/or positive MTB biopsy culture and/or histology in keeping with MTB infection used as a reference. PPV: positive predictive value, NPV: negative predictive value, CI: confidence interval. HIV: human immunodeficiency virus. *: p=0.031 (Chi squared).

Table E5: Per sample diagnostic accuracy of the XpertMTB/RIF assay for the diagnosis of pleural tuberculosis, using either fluid or biopsy culture as reference standard. Stratified by HIV status.

| Sample type | Patients | Fluid culture positive vs. fluid culture negative | | | | Biopsy culture positive vs. biopsy culture negative | | | |
| --- | --- | --- | --- | --- | --- | --- | --- | --- | --- |
|  |  | Sensitivity%  (95% CI) | Specificity%  (95% CI) | PPV%  (95% CI) | NPV%  (95% CI) | Sensitivity%  (95% CI) | Specificity%  (95% CI) | PPV%  (95% CI) | NPV%  (95% CI) |
| Fluid | All patients | 31.3% (14.2, 55.6)  5/16 | 90% (79.9, 95.4)  54/60 | 45.5% (21.3, 72)  5/11 | 83.1% (72.2, 90.3)  54/65 | 23.1% (11.1, 42.1)  6/26 | 96.2% (87.1, 99)  50/52 | 75% (41, 92.9)  6/8 | 71.5% (60, 80.7)  50/70 |
|  | HIV un-infected | 16.7% (3.1, 56.4)  1/6 | 92.2% (79.3, 97.3)  35/38 | 25% (4.6, 70)  1/4 | 87.5% (73.9, 94.6)  35/40 | 11.8% (3.3, 34.4)  2/17 | 96.7% (83.4, 99.5)  29/30 | 66.7% (20.8, 93.9)  2/3 | 66% (51.2, 78.2)  29/44 |
|  | HIV infected | 60% (23.1, 88.3)  3/5 | 50% (15.1, 85)  2/4 | 60% (23.1, 88.3)  3/5 | 50% (15.1, 85)  2/4 | 50% (15.1, 85)  2/4 | 100% (43.9, 100)  3/3 | 100% (34.3, 100)  2/2 | 60% (23.1, 88.3)  3/5 |

PPV: positive predictive value, NPV: negative predictive value, CI: confidence interval. HIV: human immunodeficiency virus.

Table E6: Non-TB patients with ADA and/or INF-γ levels above specified cut points

| High levels ADA | | |
| --- | --- | --- |
| Patient | Levels ADA (IU/L) | Final Diagnosis |
| 11 | 66 | Systemic lupus erythematosus- not treated for TB but for bacterial infection |
| 26 | 111.8 | Empyema, Streptococcus Group B |
| 89 | 48.8 | Empyema |
| High levels INF-γ | | |
| Patient | Levels INF-γ (pg/ml) | Final Diagnosis |
| 84 | 5.025 | Lost to follow up |
| 89 | 9.955 | Empyema |

INF-γ: Interferon gamma. ADA: Adenosine deaminase. INF-γ cut point of 107·7 pg/ml determined by ROC. ADA cut point of 30 U/l is used for clinical decision-making at Groote Schuur Hospital.
